# Supplementary material for: Determining Pneumocystis jirovecii Colonisation from Infection Using PCR-Based Diagnostics in HIV-Negative Individuals
Source: Diagnostics (Basel). 2024 Jan 4;14(1):114. doi: 10.3390/diagnostics14010114 (PMC10802892; doi:10.3390/diagnostics14010114)
Supplement: Supplementary file 1 [file diagnostics-14-00114-s001.zip › diagnostics-2774001-supplementary.pdf]

Supplementary data

- Immunosuppressive drugs recorded: any chemotherapy, alkylating agents, anthracyclines, tumour necrosis factor inhibitors, methotrexate, rituximab, anti-metabolites, leflonamide, hydroxyurea, sulfasalazine, mycophenolate, calcineurin inhibitor, immunotherapy, Tyrosine kinase inhibitors, mammalian target of rapamycin (mTOR) inhibitors
- Autoimmune diseases recorded: collagenous colitis, rheumatoid arthritis, dermatomyositis, lichen planus, membraneous glomerulonephritis, ulcerative colitis, microscopic polyangiitis, inflammatory myopathy, lupus, giant cell arteritis, bullous pemphigoid, paraneoplastic pemphigus, autoimmune haemophilia
- Data set

Definite infection

Sex	Age	Deceased D+30	Haem. cancer	Non-haem. cancer	Solid organ transplant	Chronic lung disease	Haemo-dialysis	Diabetes	Chronic liver disease	Autoimmune disease	Immunosuppressive drug prescribed	Steroids ≥ 2 wks >15mg/day	Prophylax	Fever	Dyspnoea	Cough	Hypoxia (< 95%)	Bacterial	Viral	Other infective	Non-infect	Ground glass	Reticular opacities	Nodules	Consolidation	Other contributing pathology	Sample type	qPCR
M	63	0	1	0	0	0	0	0	0	0	1	0	0	1	1	0	1	0	0	0	0	1	0	0	0	1	BAL	1.2x10^5
M	69	0	0	1	0	0	0	0	0	0	1	1	0	1	1	0	0	0	0	0	0	1	0	0	0	0	BAL	7.7x10^5
M	56	0	1	0	0	0	0	0	0	1	1	0	0	1	1	1	0	0	0	0	0	1	0	1	0	0	BAL	3.5x10^5
M	37	0	1	0	0	0	0	0	0	0	1	0	1	1	1	1	1	0	0	0	0	1	0	0	0	0	BAL	1.4x10^5
M	57	0	1	0	0	1	0	0	1	0	1	0	1	1	1	0	0	0	0	0	0	0	0	0	0	1	BAL	3.2x10^5
F	51	1	0	1	0	0	0	0	0	0	0	0	0	1	1	1	1	0	0	0	0	0	0	0	0	1	BAL	1.7x10^6
F	38	0	0	0	0	0	0	1	0	1	0	1	0	1	1	1	1	0	0	0	0	0	0	0	1	1	BAL	1.9x10^7
F	47	0	0	0	0	1	0	0	0	0	0	0	0	0	1	0	0	0	0	0	0	1	0	1	0	1	BAL	8.2x10^3
F	53	0	0	1	0	0	0	0	0	0	1	1	0	1	0	0	0	0	0	0	0	1	0	0	1	0	BAL	1.9x10^4
M	63	1	1	0	0	0	0	1	0	1	1	1	0	0	1	1	1	0	0	0	0	0	0	0	1	0	BAL	4.8x10^4
F	76	0	0	1	0	0	0	0	0	0	1	0	0	0	1	1	0	0	0	0	0	1	0	0	0	1	Sputum	3.6x10^4
M	76	1	1	0	0	1	0	0	0	0	1	0	0	1	1	1	1	0	0	0	0	1	1	0	1	0	Sputum	3.4x10^6
F	44	0	0	0	0	1	0	0	0	1	1	0	0	1	1	1	1	0	0	0	0	1	0	0	1	1	Sputum	2.5 x 10^5
F	56	0	0	0	0	1	0	0	0	0	0	0	0	0	1	1	1	0	0	0	0	1	0	1	1	1	Sputum	2.0x10^4
M	64	0	1	0	0	0	0	0	0	0	1	0	1	1	0	1	0	0	0	0	0	1	0	0	1	1	Sputum	1.0x10^5
F	70	0	1	0	0	0	0	0	0	0	1	0	0	1	0	0	1	0	0	0	0	1	0	0	0	1	Sputum	1.0x10^5
M	68	1	0	0	0	1	0	0	0	1	1	0	0	1	1	1	1	0	0	0	0	1	0	1	1	1	Sputum	2.5x10^6
F	53	0	1	0	0	0	0	0	0	0	1	0	1	1	1	0	1	0	0	0	0	1	0	0	1	1	Sputum	4.6x10^6
F	18	0	1	0	0	0	0	1	0	0	1	0	1	0	1	0	0	0	0	0	0	1	0	0	0	1	Sputum	6.9x10^4
F	61	0	0	1	0	1	0	0	0	0	1	0	0	1	0	1	0	0	0	0	0	1	0	0	1	0	Sputum	1.5x10^5
F	66	0	0	1	0	1	0	0	0	0	1	0	0	0	1	1	1	0	0	0	0	1	0	0	1	1	Sputum	9.0x10^5
M	63	1	0	1	0	0	0	1	0	1	1	0	0	0	1	1	1	0	0	0	0	0	0	0	1	1	Sputum	1.9x10^7
M	75	0	0	1	0	0	0	0	0	0	1	0	0	1	1	1	1	0	0	0	0	1	0	0	1	1	Sputum	2.4x10^5
M	78	0	0	1	0	1	0	1	0	1	0	1	0	1	1	1	1	0	0	0	0	1	0	1	1	1	Sputum	5.3x10^5
M	87	0	0	0	0	0	0	1	0	1	0	1	0	0	1	1	1	0	0	0	0	0	0	0	1	1	Sputum	6.9x10^6
M	74	0	0	1	0	0	0	0	0	0	0	1	0	0	1	0	1	0	0	0	0	0	0	1	0	1	Sputum	3.1x10^7
M	77	0	1	1	0	0	0	0	0	0	0	1	0	1	1	0	1	0	0	0	0	0	0	0	1	1	Sputum	2.6x10^6
M	74	0	1	0	0	1	0	0	0	0	0	0	0	0	1	1	1	0	0	0	0	0	0	0	0	1	Sputum	2.7x10^4

Possible infection

Sex	Age	Deceased D+30	Haem. cancer	Non-haem. cancer	Solid organ transplant	Chronic lung disease	Haemo-dialysis	Diabetes	Chronic liver disease	Autoimmune disease	Immunosuppressive drug prescribed	Steroids ≥ 2 wks >15mg/day	Prophylax	Fever	Dyspnoea	Cough	Hypoxia (< 95%)	Bacterial	Viral	Other infective	Non-infect	Ground glass	Reticular opacities	Nodules	Consolidation	Other contributing pathology	Sample type	qPCR
F	30	1	1	0	0	0	0	0	0	0	1	1	1	0	1	1	1	1	0	0	1	1	0	0	1	1	BAL	3.0x10^3
M	65	1	1	0	0	1	0	1	0	0	1	0	0	1	1	0	0	0	0	0	1	0	0	0	0	1	BAL	3.7x10^3
M	62	0	1	0	0	0	0	0	0	0	1	0	0	1	1	1	0	1	1	0	1	0	0	1	0	0	BAL	5.4x10^3
M	42	0	0	0	0	0	0	1	1	0	0	0	0	0	1	0	1	0	1	0	0	0	0	0	1	1	BAL	2.5 x 10^5
M	69	1	0	0	0	1	0	0	0	1	1	0	0	1	1	1	0	1	0	0	1	0	0	0	0	1	BAL	8.9x10^3
M	54	0	1	0	0	0	0	0	0	0	1	0	0	1	1	1	1	0	0	0	1	1	0	1	0	1	BAL	1.2x10^4
F	58	0	1	0	0	0	0	1	0	0	1	0	1	1	0	0	0	1	0	1	0	1	0	0	0	1	BAL	1.5x10^4
M	77	1	0	1	0	0	0	0	0	0	0	0	0	1	1	1	1	1	0	0	1	1	0	0	1	1	BAL	4.5x10^4
M	73	0	0	1	0	0	0	0	0	0	0	1	0	0	1	1	1	1	0	0	0	0	0	1	0	1	BAL	5.4x10^4
M	62	0	1	0	0	0	0	0	0	0	1	0	0	0	1	1	1	0	0	0	1	0	0	1	1	1	BAL	2.8x10^5
M	60	0	0	0	0	0	0	0	1	1	1	0	0	1	1	1	1	1	0	1	0	0	0	0	1	0	BAL	1.8x10^6
M	69	0	1	0	0	0	0	0	0	0	1	1	1	1	1	0	1	0	1	0	0	1	0	0	1	0	BAL	2.1x10^6
M	68	0	0	1	0	1	0	0	0	0	1	1	0	1	1	1	1	1	0	0	0	0	0	0	1	1	BAL	3.7x10^7
F	71	0	0	1	0	1	0	0	0	0	1	1	0	0	1	1	1	1	1	0	0	1	0	0	1	1	Sputum	1.6x10^4
F	79	0	0	1	0	1	0	0	0	0	1	0	0	1	1	0	1	0	0	0	1	1	0	0	0	1	Sputum	1.8x10^4
F	52	0	0	1	0	0	0	0	0	0	1	1	1	0	1	0	0	0	0	1	0	1	0	0	0	1	Sputum	3.5x10^4
F	61	0	0	1	0	1	0	0	0	0	1	0	0	0	1	1	1	0	0	0	1	0	0	0	1	1	Sputum	4.3x10^4
M	73	0	0	0	0	0	0	0	0	1	0	1	0	0	1	1	1	1	0	0	0	0	0	0	1	0	Sputum	4.9x10^4
F	67	0	1	0	0	0	0	0	0	0	1	0	0	1	1	1	1	0	0	0	1	1	0	0	1	0	Sputum	9.3x10^4
M	66	1	0	1	0	0	0	1	0	0	1	0	0	0	1	1	1	0	1	0	0	1	1	0	0	1	Sputum	1.1x10^5
M	62	0	0	1	0	1	0	0	0	0	1	0	0	1	1	1	1	1	0	0	0	1	0	0	0	1	Sputum	1.2x10^5
F	75	0	1	0	0	0	0	1	0	0	1	1	0	1	1	1	1	0	1	0	0	1	0	0	0	1	Sputum	1.4x10^5
M	76	1	0	0	0	1	0	0	0	1	1	0	0	0	1	1	1	0	0	0	1	0	0	0	0	1	Sputum	1.7x10^5
F	72	0	0	0	0	0	0	0	0	1	1	1	0	0	1	1	1	0	1	0	0	1	0	0	0	0	Sputum	2.9x10^5
M	65	1	0	1	0	1	0	0	0	0	0	1	0	1	1	1	1	0	0	0	1	1	0	0	0	1	Sputum	5.7x10^5
M	73	0	0	1	0	1	0	0	0	0	1	0	0	0	1	1	1	0	0	0	1	1	0	1	1	1	Sputum	9.9x10^5
M	64	0	0	1	0	1	0	0	0	0	1	0	0	0	1	0	0	0	0	0	1	1	0	1	0	1	Sputum	1.0x10^6
F	75	0	0	1	0	0	0	0	0	0	1	0	0	0	1	0	1	1	1	0	0	0	0	0	0	1	Sputum	5.9x10^6
M	77	1	0	1	0	0	0	0	0	0	1	0	0	0	1	1	1	0	1	0	1	1	0	0	0	1	Sputum	1.6x10^7
M	70	0	0	1	0	1	0	0	0	0	0	1	0	0	1	1	1	0	0	0	1	1	0	0	0	1	Sputum	4.6x10^7

Colonisation

Sex	Age	Deceased D+30	Haem. cancer	Non-haem. cancer	Solid organ transplant	Chronic lung disease	Haemo-dialysis	Diabetes	Chronic liver disease	Autoimmune disease	Immunosuppressive drug prescribed	Steroids ≥ 2 wks >15mg/day	Prophylax	Fever	Dyspnoea	Cough	Hypoxia (< 95%)	Bacterial	Viral	Other infective	Non-infect	Ground glass	Reticular opacities	Nodules	Consolidation	Other contributing pathology	Sample type	qPCR
M	22	0	1	0	0	1	0	0	0	0	1	0	1	0	1	0	1	0	1	0	1	0	0	1	1	0	BAL	2.5 x 10^5
M	34	0	0	0	1	0	0	0	0	0	1	0	0	0	0	1	0	0	1	1	0	0	0	1	0	1	BAL	4.3x10^4
M	52	0	0	0	0	1	0	1	0	0	0	0	0	0	1	1	0	0	0	0	1	0	0	1	0	1	BAL	2.2x10^4
M	61	0	0	0	0	1	0	0	0	0	0	0	0	0	1	0	0	1	1	1	0	0	0	1	0	1	BAL	8.2x10^3
F	68	0	0	0	0	0	0	0	0	1	1	0	0	0	0	1	0	0	0	0	1	0	0	0	0	1	BAL	2.5 x 10^5
M	70	0	1	0	0	1	0	1	0	0	1	0	0	1	0	0	0	0	0	0	1	0	0	0	1	1	BAL	2.0x10^5
F	25	0	1	0	0	0	0	0	0	0	1	1	1	0	1	0	0	0	0	0	1	0	0	0	0	1	Sputum	1.3x10^4
M	36	0	0	0	0	0	0	0	1	0	0	0	0	0	1	1	1	1	0	1	1	0	0	0	1	1	Sputum	1.4x10^6
M	37	0	1	0	0	0	0	0	0	0	0	1	0	1	1	0	0	0	0	0	1	1	0	0	1	0	Sputum	6.6x10^4
M	40	0	0	0	0	0	0	0	0	0	0	0	0	0	1	0	1	0	0	0	1	0	0	0	0	1	Sputum	3.0x10^4
F	43	0	0	0	0	0	0	0	0	1	1	1	0	0	1	1	0	0	0	0	1	0	0	0	1	0	Sputum	1.4x10^4
F	44	0	0	1	0	0	0	0	0	0	1	0	0	1	1	1	0	0	0	0	0	1	0	0	1	0	Sputum	3.8x10^4
M	54	0	0	0	0	1	0	1	0	1	0	1	0	0	1	1	1	0	0	0	1	0	0	0	0	1	Sputum	2.0x10^5
F	57	0	0	1	0	0	0	0	0	0	1	0	0	0	1	1	0	0	0	0	0	0	0	0	0	1	Sputum	2.5 x 10^5
F	57	0	0	0	0	1	0	0	0	0	0	0	0	0	1	1	1	0	0	0	1	0	0	0	1	1	Sputum	4.8x10^4
F	59	0	0	1	0	0	0	1	0	0	1	0	0	1	0	1	1	0	0	0	0	0	0	0	1	1	Sputum	1.3x10^5
M	59	0	0	1	0	0	0	0	0	0	0	0	0	0	1	1	1	0	0	0	1	1	0	1	1	1	Sputum	1.2x10^5
M	63	0	1	0	0	0	0	0	0	0	1	0	0	0	1	0	0	0	0	0	1	1	0	0	0	1	Sputum	6.2x10^4
M	66	1	0	1	0	1	0	0	0	0	0	0	0	1	1	1	1	1	0	0	1	1	0	1	0	1	Sputum	2.5 x 10^5
M	67	1	0	1	0	0	0	0	0	0	1	0	0	0	0	0	0	0	0	1	1	0	0	0	1	1	Sputum	6.5x10^5
M	72	0	0	0	0	1	0	0	0	1	1	0	0	0	1	1	1	0	0	0	1	0	0	0	0	1	Sputum	2.5 x 10^5
M	82	0	0	0	0	1	0	0	0	1	0	1	0	0	0	1	0	0	1	0	1	0	0	0	1	1	Sputum	2.5 x 10^5
M	85	1	0	1	0	1	0	0	0	0	0	1	0	0	1	1	1	0	0	0	1	0	0	0	0	1	Sputum	3.9x10^5
M	55	0	1	0	0	0	0	0	0	0	1	0	0	0	1	1	0	0	1	0	0	0	0	0	1	0	Sputum	1.3x10^4
